# Sustainability, Circularity, and Innovation in Wood-based Panel Manufacturing in the 2020s: Opportunities and Challenges

**DOI:** 10.1007/s40725-024-00229-1

**Published:** 2024-08-17

**Authors:** Rosilei Garcia, Ingrid Calvez, Ahmed Koubaa, Véronic Landry, Alain Cloutier

**Affiliations:** 1 Renewable Materials Research Centre (CRMR), Faculty of Forestry, Geography, and Geomatics, Université Laval, Québec, QC Canada; 2 Forest Research Institute, Université du Québec en Abitibi-Témiscamingue (UQAT), Rouyn-Noranda, QC Canada; 3 Department of Wood and Forest Sciences, Faculty of Forestry, Geography, and Geomatics, Université Laval, 2425 De La Terrasse Street, Québec, QC G1V 0A6 Canada

**Keywords:** Logging residues, Fire-damaged wood, Recycled wood, Lightweight panels, Multifunctional panels, Antimicrobial laminates

## Abstract

**Purpose of review:**

This review explores the opportunities and challenges associated with using unconventional and underutilized wood sources, such as fast-growing species, logging residues, fire-damaged wood, and post-consumer wood, to manufacture wood-based composite panels (WBCPs), particularly particleboard, medium-density fiberboard (MDF), and oriented strand board. This paper also discusses recent advancements in lightweight and multifunctional panels, with new features such as fire resistance, electrical conductivity, electromagnetic shielding, and antibacterial laminates.

**Recent findings:**

Climate change, wildfires, and competition from the energy sector threaten current sources of fiber supply for WBCP manufacturing in some regions. Logging residues are abundant but underutilized in some areas, and the abundance of fire-damaged wood is expected to increase in the coming years due to climate change. These raw materials’ effects on panel properties and technological limitations are discussed. Recycled wood is increasingly used for non-structural panels, but challenges remain when it comes to recycling panels, particularly post-consumer MDF. Conventional and emerging materials used in lightweight and multifunctional panels are also presented. Natural substances like cellulose, nanocellulose, chitosan, lignin, protein, and phytic acid are promising alternatives to conventional fire retardants. Innovative products such as MDF that contains carbon-based conductive fibers and antimicrobial laminates that use green-synthesized metal compounds are also reported.

**Summary:**

This review shows that the WBCP industry can improve its sustainability by optimizing and diversifying wood sources, better managing and recycling post-consumer panels, and using more environmentally friendly materials. The hazardous chemicals in adhesives, fire retardants, and coatings are the main obstacles to recycling panels and creating a more circular economy within the WBCP industry.

## Introduction

**Fig. 1:**
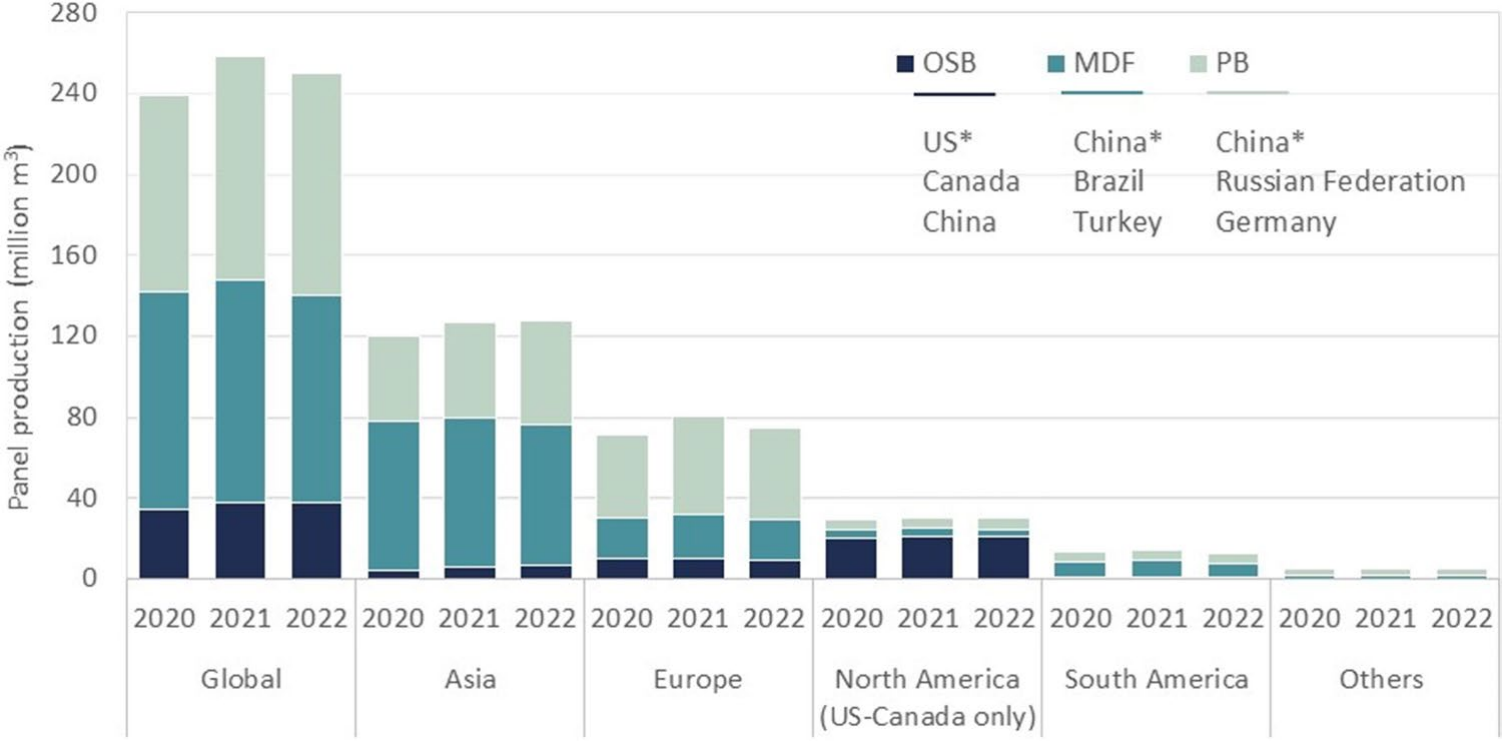
Global and regional PB, MDF, and OSB production, 2020–2022. * The top three producing countries are listed for each panel type. The authors generated the figure using the FAO’s FAOSTAT database [2]

Wood-based composite panels (WBCPs) are essential to the sustainable development of the forest industry and make a significant social, economic, and environmental contribution to it. According to the latest Food and Agriculture Organization of the United Nations (FAO) report [1], the wood and WBCP manufacturing industry employed 19.4 million people worldwide between 2017 and 2019, which corresponds to over 58% of total employment in the forest sector. Global WBCP production reached record levels in 2021–2022. In 2022, it hit 375.3 million m^3^, with particleboard (PB), medium-density fiberboard (MDF), and oriented strand board (OSB) accounting for 66.7% of that amount. PB had the highest production, at over 110 million m^3^ in 2022, followed by MDF, with a total output of about 102 million m^3^. Asia and Europe are the largest producers of PB and MDF. North America is the largest producer of OSB and produced over 20 million m^3^ of it in 2022, which represents 54.6% of global production. Europe comes in second, with about 25% of production, followed by Asia, with 18.7% (see Fig. 1) [2]. WBCPs have also been essential for valorizing wood residues and lower-value wood and storing carbon for decades. WBCPs used in furniture can store carbon for around 25 to 30 years, while those used in buildings can store carbon for 50 + years, until the panels are recycled or disposed of [3]. A study by Puettman [3] showed that WBCPs produced in North America store far more carbon than they release in manufacturing. The study estimated that they stored 354 million metric tons of carbon dioxide equivalent (CO_2e_) in 2019 alone.

WBCPs are produced from various raw materials depending on regional resource availability. Canadian OSB manufacturers use mainly small-diameter trembling aspen and white birch trees, with minimal incorporation of softwoods. Conversely, Southern yellow pine species dominate OSB manufacturing in the United States (US), with a small proportion of other species, such as birch, maple, or sweetgum, also used [4]. OSB is produced from softwood (pine and spruce) and hardwood (aspen) in Europe, while rubberwood is commonly used in Asia. PB and MDF are widely made from low-value or fast-growing wood species, sawmill residues (chips, sawdust, and shavings), and an increasing proportion of recycled wood and non-wood lignocellulosic materials such as flax, hemp, bamboo, and bagasse [5, 6]. Eucalyptus is the most widely planted fast-growing hardwood species in the world. Its fibers are particularly advantageous for manufacturing MDF as they are short and obtained through steam explosion, a process known to produce better-quality fibers than that produced by the thermomechanical pulping process, which is commonly used in North America and Europe. These high-quality fibers give the products they are incorporated in excellent properties and outstanding surface quality. Brazil and China have the largest and second-largest eucalyptus plantation areas in the world, which represent 22% and 20% of overall planted area, respectively [7]. These countries have thus become leading MDF-producing countries, as Fig. 1 shows. Canadian PB and MDF producers commonly use sawmill residues as raw material and are well-integrated into forest sector supply chains. However, recent studies suggest that their fiber supply could decline considerably in the coming years due to climate change, environmental and social issues, clean energy policies, and competition from other sectors [8, 9]. Climate change could reduce the availability of commonly used tree species, increase the frequency and intensity of forest disturbances, and upend the current supply chain. In 2023, the scale of wildfires in Canada prompted authorities to recommend a 15% reduction in allowable annual cut, which corresponds to 619,200 m^3^ of wood per year, for 2023–2028 in the province of Quebec alone [10]. The impact that these measures will have on the panel industry's raw material supply has yet to be assessed. Furthermore, Canadian federal government policies, such as the Clean Fuel Standard to reduce carbon emissions by 2030, may increase the level of competition for wood residues between the WBCP industry and the bioenergy sector [8, 9]. The Russian Federation is a large PB producer and has the most forest area in the world (20% of all forest area on Earth). However, its forests are also vulnerable to intense wildfires and insect damage. It is estimated that forest fires affect three to four times as much forest area in the Russian Federation as is legally or illegally harvested each year [11]. In Europe, wood waste management and recovery are crucial environmental issues, and post-consumer wood is an essential source of raw material for PB production. The European Parliament recently endorsed intensifying the use of cascaded wood to promote the circular economy and mitigate climate change. The cascade approach involves reusing wood in multiple products before utilizing it for energy [12]. This approach supports the European panel industry since PB and MDF constitute the primary products of cascading wood valorization. However, implementing more efficient recycling technologies such as sorting, fragmentation, pretreatments, and decontamination technologies to increase the proportion of post-consumer wood in mills represents a challenge [13]. All these issues regarding current raw material sources have led to the search for alternative raw materials from unconventional or underutilized sources or recycled wood to ensure fiber supply, productivity, and circularity from a sustainable development perspective.

Moreover, the panel industry faces a significant challenge in terms of developing innovative, efficient, and environmentally friendly products to stay competitive in a market in which consumers are more environmentally conscious and demanding while also adhering to government regulations and laws. Among the new advances, there is growing interest in creating lightweight and multifunctional panels using more efficient processes and non-hazardous, bio-based, and biodegradable materials while considering opportunities for recycling post-consumer panels into new products. Laminate surface technologies for PB and MDF furniture and flooring are another area of ongoing innovation. One of the latest advances concerns antimicrobial laminates for applications in which high hygiene levels are required, such as kitchens and worktops in public and hospital settings [14, 15].

In this paper, we address two main topics. First, we explore the latest research on diversifying or optimizing new sources of raw materials for manufacturing PB, MDF, and OSB. These sources include alternative wood species, logging residues, fire-damaged wood, and post-consumer wood, especially from PB and MDF. Second, we present recent product advances, key materials, properties, and challenges, specific to lightweight and multifunctional panels. Multifunctional panels include panels with new functionalities such as fire resistance, electrical conductivity, electromagnetic shielding, and antimicrobial laminate surfaces.

## New Sources of Raw Materials

### Alternative Wood Species

OSB was created in the 1960s, initially for underutilized wood species, such as low-grade hardwoods like aspen and poplar in Canada. The widespread adoption of OSB in residential construction, especially in North America, along with increasing demand for these panels, prompted researchers to investigate the use of other species for OSB production. Researchers are currently exploring producing OSB from various types of wood species, including softwoods in Canada and unconventional fast-growing species worldwide [16–19]. In Canada, OSB manufacturers typically add only 10–15% softwood to the manufacturing process despite the abundance of softwood species. Thus, small-diameter softwood logs that are discarded by the lumber industry could be an alternative source of raw material for the OSB industry. According to Zhuang et al. [16, 17], Canadian softwood species such as black spruce, balsam fir, jack pine, and aspen-black spruce blends are feasible options for OSB manufacturing. However, in their study, the OSB made from softwood exceeded the 10% maximum thickness swelling requirement specified in CSA standard O437-93 (R2011). Also, Zhuang et al. [17] found that OSB made from black spruce had higher internal bonding (IB) strength than that made from aspen, but aspen provided better thickness swelling and bending properties. These studies indicate that species proportions and mixtures need to be optimized and processes, such as flaker cutting parameters and strand features, must be better adapted to ensure all required specifications are met. One innovative approach that has been considered is to develop a strander-canter machine with a new cutterhead design to produce good-quality strands as a co-product of jack pine log processing for lumber [18]. Lunguleasa et al. [19] took a different approach and compared the physical and mechanical properties of OSB made from two types of wood: mixed softwood (fir-spruce-pine), which is frequently used in Europe, and fast-growing hardwood (poplar-willow-birch). OSB produced from fast-growing hardwood had better mechanical properties and exhibited slightly less thickness swelling than that made from mixed softwood. Based on these findings, the authors recommend using fast-growing hardwood species for OSB production in Europe instead of softwood species to avoid the over-exploitation of softwoods. Also, Lunguleasa et al. [20] investigated the strand features, such as slenderness ratio and specific surface area of four wood species: spruce, pine, poplar, and willow. Willow and poplar produced the best strand quality and OSB properties. These findings open up possibilities for several alternative wood species to be utilized for OSB manufacturing.

PB and MDF are more tolerant than OSB when it comes to raw material quality, which means that a broader range of fast-growing species can be used for them than for OSB, depending on the region. Lesser-known or underutilized wood species that can be suitable for PB include athel, Eastern redcedar, peterebi, kiri, willow, kadam, mangrove tree, date palm, and sengon [6]. For instance, studies have shown that willow species, specifically *Salix viminalis*, can provide a cost effective and suitable fiber source for PB and MDF. Sean and Labrecque [21] demonstrated that up to 30% of the conventional raw materials used in those panels can be replaced with three-year-old willow stems (with or without bark) without compromising the physical and mechanical properties of the panels. Moreover, willow leaves and bark contain active compounds that have antimicrobial, analgesic, antioxidant, anticarcinogenic, and antidiabetic properties and could therefore be used to produce bioproducts for pharmacological and medicinal purposes [22]. In Canada, willows have a growth rate potential that is four to six times greater than that of commercially cultivated species, which has led the federal government to establish incentive programs to encourage planting them [23, 24]. Rahman et al. [25] report that Malaysia is currently facing a shortage of rubberwood, commonly used in WBCP, due to the replacement of rubberwood plantations by more profitable oil palm plantations for purposes other than panel production. To counter this shortage, Malaysia and other Asian countries are investigating several fast-growing species as potential alternatives to rubberwood for the panel industry. One of these species is *Leucaena leucocephala*. In addition to its fast growth and successful trial plantings, PBs produced from 18-month-old *Leucaenae* trees have shown good properties. Additionally, other species, such as short-rotation eucalyptus and cottonwood, are being considered for MDF production in the Southeastern US [26].

### Logging Residues

Forestry operations produce large quantities of logging residues annually. Although leaving some of these residues can help to maintain soil fertility, site productivity, carbon sinks, and biodiversity (30–70% depending on site quality), a considerable proportion of them could be collected and utilized [27, 28]. A portion of these residues is used for energy recovery, but most remain on the cutblocks or along roadsides and therefore represent a potential source of degradation and greenhouse gas emissions and a fire hazard [11, 29]. The practice of logging residue removal varies both between and within countries, influenced by their policies, regulations, and certifications [28]. In Finland, logging residues are widely utilized, mainly for energy, with 30% left at the logging site to ensure nutrients remain in the forest [30]. Conversely, industry practices in British Columbia, Canada, often involve leaving logging residues to decompose in the cutting area or burning them in the open using slash-pile burning practices. While these cost-effective methods meet minimum management requirements, they generate significant greenhouse gas emissions and release harmful particulates, exacerbating climate change and adversely affecting human health [31]. Countries like the Russian Federation, Canada, and the US, which have vast forest areas (20.1%, 8.6%, and 7.6% of global forest area, respectively), generate a significant amount of unused logging residues [11, 27, 28, 32]. Pokharel et al. [27] found that only 4% of mills in the Southern US utilized logging residues for energy production despite the abundance of this feedstock and its proximity to mills. In Canada, the average mass of residues generated per hectare logged is about 26 ± 16 oven-dry tons/ha, and the annual availability of residues is estimated at 21 million oven-dry tons [33]. Desrochers et al. [34] reported that Quebec alone generated 7.1 million oven-dry tons of logging residues (trunks, tops, and branches) in 2019. Around 2.3 million oven-dry tons of residues were expected to remain in the forest to maintain soil fertility and comply with Quebec's sustainable forest management laws and regulations. Only 0.1 million oven-dry tons went to paper mills, cogeneration, and energy product plants. These data indicate that the utilization rate of logging residues in Quebec is less than 2% and that there is an abundance of residues available with a potential for valorization as biomaterials or bioproducts such as WBCPs. The panel industry can benefit significantly by making use of these residues, which would reduce its dependence on sawmill residues, help it secure a supply of fiber from an abundant raw material, and contribute to better use of forest resources. However, recovering these residues can be challenging due to a variety of factors, such as site accessibility, geographical distances, transport costs, material heterogeneity in terms of composition, shape, size, or species mix, and cost and adaptability of shredding equipment to process heterogeneous materials [35]. Many feasibility studies have assessed the availability of logging residues and their proximity to mills in an effort to overcome these challenges and explore potential valorization pathways [27, 33]. A possible drawback for the Canadian panel industry is competition from the bioenergy sector, which is set to grow because of government policies promoting clean energy, that could lead to a supply shortage for the panel industry by 2030 [8].

Few recent studies have examined using logging residues in panel manufacturing [36–38]. Şahin [36] evaluated the feasibility of using three types of forest residues – pine needle litter, mixed oak-hornbeam broadleaves, and pinecones – to manufacture PB bonded with urea–formaldehyde (UF) resin. Logging residues were combined with wood particles to form the core layer of the PB with proportions ranging from 10 to 50%, while the surface layers comprised 100% wood particles. The residues exhibited a low pH and high solubility in hot water, cold water, and 1% NaOH, possibly due to their high extractive and resin acid content. These chemical features negatively impact the properties of panels bonded with UF resin. The PB panels that contained residues had higher water absorption values, but when the residues were present in low proportions (10–20%), the panels met the applicable standards for modulus of elasticity (MOE), modulus of rupture (MOR), and IB strength. Pędzik et al. [37] assessed the impact that logging residues from Scots pine branches have on the properties of PB bonded with melamine-urea–formaldehyde (MUF) resin. The branch-based PB panels exhibited greater IB strength, lower MOE values, and more thickness swelling than the panels made of stem wood particles. Despite this, all the panels met the requirements for indoor use. Vititnev et al. [38] examined the chipping and refining parameters of logging residues used in wet-process fiberboard manufacturing. They reported that adapting a new disc refiner plate geometry for different grades and sizes of logging residues (branches and tops) improved fiber quality and panel properties.

### Fire-damaged Wood

Although research on the use of fire-damaged wood for WBCP production is currently scarce, the unprecedented scale of recent wildfires worldwide has underscored the significance of this topic. Between January and May 2022, wildfires burned 3.8 million hectares in Russia, 400,000 hectares more than for the same period in 2021, a year in which a record high of over 18 million hectares of forest burned [11]. According to the Canadian Interagency Forest Fire Centre, between January and November 2023, Canada also had over 18 million hectares of forest burned [39]. Anttila and Verkerk [40] reported that the frequency and severity of forest disturbances, including fires, may worsen with climate change and threaten forest productivity and fiber supply. As a result, panel factories are expected to incorporate more and more fire-damaged wood in their supply.

Only a few studies have investigated using fire-damaged wood for panel manufacturing. One such study, by Moya et al. [41], investigated the effect the level of fire damage (light, moderate, or severe) and the amount of red pine wood bark content (0–20%) have on OSB properties. OSB produced from burnt wood without bark had similar properties as OSB from unburnt wood, regardless of the fire damage level. Increasing the bark content reduced the MOR and MOE due to the small size and low strength of bark particles. However, bark also reduced thickness swelling because of the bark’s hydrophobic properties, which were enhanced with fire exposure due to the breakdown of hemicelluloses (the most hygroscopic polymer) and a relative increase in lignin content (the most hydrophobic polymer). Another study, by Akgül et al. [42], analyzed using mixtures of burnt pine wood from a fire-impacted area in Turkey and unburned wood (50% beech and 50% oak) in MDF manufacturing. The panels were manufactured under industrial conditions and bonded with UF adhesive. The results showed that IB strength did not change significantly while bending properties and screw withdrawal resistance increased. However, burnt wood negatively impacted thickness swelling and Janka hardness. Additionally, it increased the average surface roughness of the MDF. Since MDF surfaces should be smooth and not very absorbent to provide better painting and finishing performance, this aspect should be explored further.

### Post-consumer Wood

The ISO/DIS 17300–1 [43] provides definitions for “post-consumer wood,” “recycled material,” and “wood waste”, which can be helpful in this section. “Post-consumer wood” refers to “*wood generated by the end-users of wood products that has fulfilled its intended purpose, including materials returned from within the distribution chain*”. “Wood waste” is “*wood that is subject to destruction (burial) due to contamination by hazardous or harmful substances, as well as when it is impossible or ineffective to use residues, waste, and used wood that is unsuitable for reuse*”. The term “wood waste” is commonly used in the context of classification and management. Lastly, “recycled material” is “*material that has been recovered, or otherwise diverted, from the waste stream, either from the manufacturing process (i.e. post-industrial recycled materials, but not in-house scrap) or after consumer use (i.e. post-consumer recycled materials), that is reused in the manufacture of new products, and for which the organization can provide evidence of compliance with the requirements of the Due Diligence System”*.

According to the FAOSTAT database [2], the amount of post-consumer wood that was recovered worldwide in 2022 was 38 million m^3^, which represents an average annual growth rate of approximately 7.8% compared to the previous two years. However, these numbers are relatively modest compared to the rate at which global demand for WBCPs is increasing, considering that these products will end up as waste in only a few decades. It is estimated that 80% of MDF (by volume) will become waste within 25 years following its production [44]. In terms of global post-consumer wood recovery, Europe is the top contributor at 82.9%, with Germany (22%), France (17.5%), and the United Kingdom (UK) (12%) being the top three contributors. The remaining 17% comes from Asia, with China accounting for 10% of global post-consumer wood recovery [2]. Post-consumer wood constitutes a sustainable resource for material recovery, particularly for PB and MDF [13, 45]. In European regions such as the UK, the panel industry is primarily responsible for recycling post-consumer wood. Soil amendment and biomass energy generation represent the second and third largest markets [45]. However, recycling wood for use in new panels is a complex process due to material heterogeneity and the presence of impurities and contaminants.

Wood waste classifications and regulations governing wood waste use in panel manufacturing vary by country. Germany classifies wood waste into four categories: AI (clean wood), AII (bonded, painted, coated, lacquered, or otherwise treated wood without organohalogens), AIII (wood waste containing organohalogens), and AIV (wood treated with hazardous preservatives, e.g., creosote, chromate copper arsenate [CCA]) – AI and AII include PB and furniture waste. AI and AII waste are accepted for panel manufacturing. AIII waste is admissible, provided the halogenated paints and coatings are largely eliminated [46]. The Waste Wood Ordinance sets out maximum permissible values for contaminants, particularly potentially toxic elements, heavy metals, organohalogens, creosote, and pentachlorophenol, that panel manufacturers must comply with [45]. France and the UK use a similar classification system with four categories: A, B, C, and D. In Canada, the classification of wood waste is determined by provincial and territorial authorities since no official regulations govern wood waste sorting [45]. In Quebec, three sorting categories are recommended for construction, renovation, and demolition (CRD) wood: Category 1 (unpainted, untreated, or unsoiled wood), Category 2 (painted, stained, or varnished wood, melamine, MDF, PB, chipboard, plywood, pallets, and veneer), and Category 3 (treated wood) [47]. Tafisa Canada is the only panel manufacturer in Quebec that uses CRD wood to make new PB (TAFIPAN®). Tafisa has devised a technology called Rewood™ that can recycle up to 244,000 tons of CRD wood annually, which makes it possible to use up to 30% CRD wood in panel production [48]. However, this recycling process works with only Category 1 CRD residues, which excludes post-consumer panels.

**Fig. 2:**
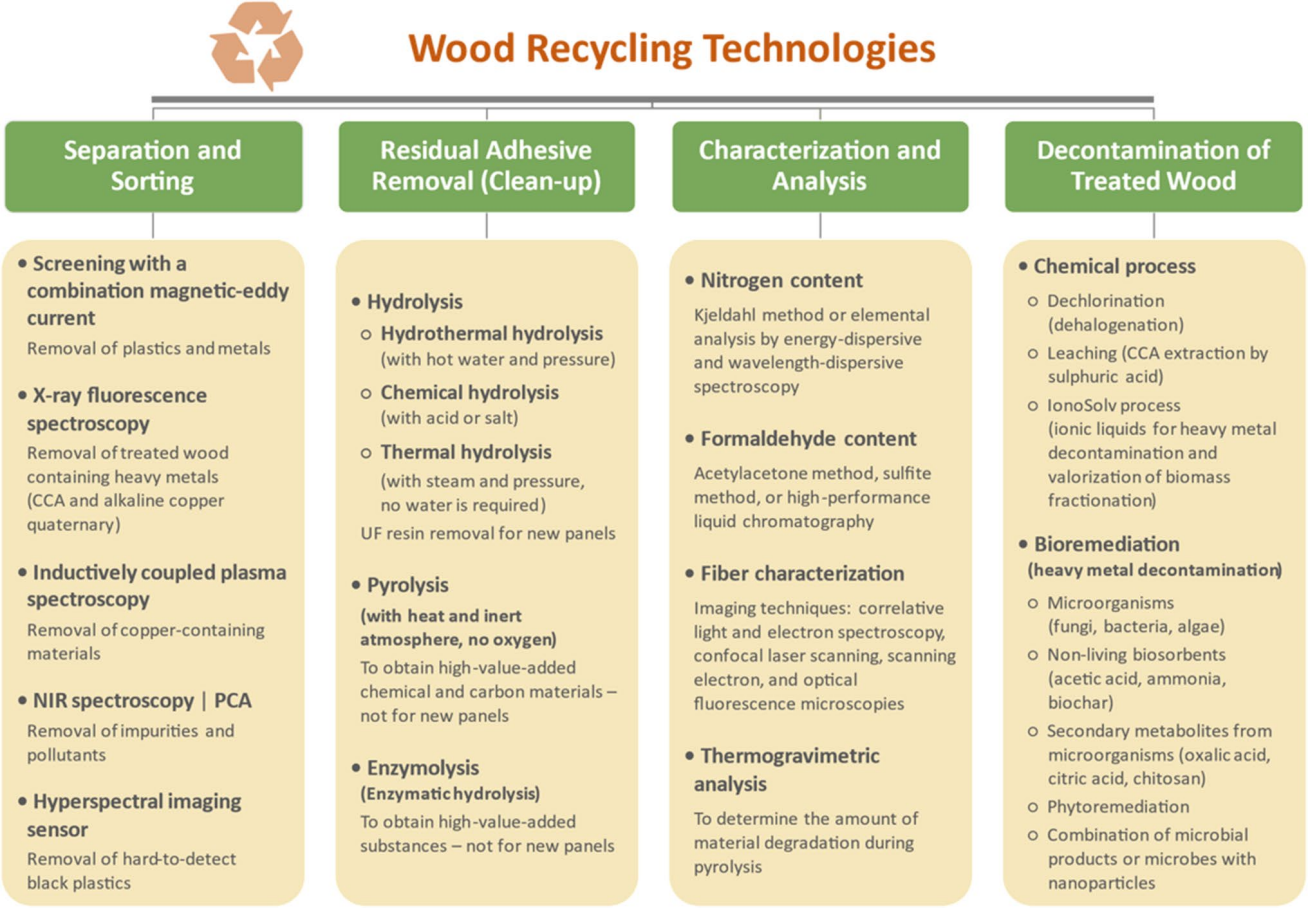
Overview of the main wood recycling technologies currently used for material recovery. For further information, refer to [13, 50–54]

Recycling technologies involve several steps, which are summarized in Fig. 2. European plants have adopted modern separation and sorting technologies by using a combination of magnetic-eddy current systems to separate metals, then automatic systems that include X-rays, near-infrared (NIR) spectroscopy combined with principal component analysis (PCA), and hyperspectral imaging sensors to eliminate impurities such as glass, stone, and plastic from wood waste. The Italian company Fantoni has successfully used this technology to recover 250,000 tons of furniture-grade wood waste to produce new MDF panels [49].

Recycling PB and MDF is a major challenge because chemical contaminants from adhesives, coatings, and additives such as preservatives and fire retardants (FRs) cannot be removed mechanically. The cured adhesive on particles recovered solely by mechanical means drastically reduces new panels' physical and mechanical properties and increases their formaldehyde and volatile organic compound (VOC) emissions [55, 56]. The cured adhesive can be eliminated through hydrolysis, pyrolysis, or enzymolysis (see Fig. 2), depending on the preferred valorization pathway. Hydrolysis has typically been employed to valorize recycled fibers by incorporating them in panels, while pyrolysis and enzymolysis have been investigated to convert WBCP waste into high-value chemicals and carbon materials [50, 57]. UF adhesive is the primary source of contamination in PB and MDF waste. However, its low moisture resistance means that it can be partly removed by hydrothermal hydrolysis, acid hydrolysis, or thermal hydrolysis. Cured UF adhesive's sensitivity to hydrolysis follows the order of acidic, then neutral, and finally, basic conditions [51]. UF hydrolysis is assessed by analyzing the solid residue (its mass loss and nitrogen content), the extract solution (its pH, nitrogen content, and formaldehyde content), and their balance using various methods, as outlined in Fig. 2. Generally, the nitrogen content attributed to UF adhesive on the recycled fibers decreases as the severity of treatment increases [58]. However, hydrolysis conditions (e.g., temperature, duration, medium pH) can also affect fiber morphology, strength, pH, and acid/base buffering capacity and ultimately interfere with the behavior of the UF adhesive used to manufacture new panels and reduce the panel’s properties [51, 58, 59]. Several studies have looked into optimizing hydrolysis conditions to find a balance between cured resin removal and fiber quality. For instance, Fu et al. [59] found the optimal thermal hydrolysis conditions to recover post-consumer PB are 140 °C for 20 min. This treatment removed around 65% of cured UF resin and made it possible to produce PB composed entirely of recycled particles that had similar properties as PB made from virgin particles.

MDF waste is more complex to recycle for new panel production than PB waste. One reason is that MDF contains more adhesive than PB. Moreover, recycled MDF fibers typically are of lower quality (shorter length, a lower slenderness ratio, and more fines than virgin fibers). These factors increase their specific surface area, which means that more adhesive is required to produce MDF panels that meet applicable standards [13, 51]. Various methods have been investigated to recover MDF fibers, including hydrothermal hydrolysis, acid hydrolysis, thermal hydrolysis, medium wave frequency, steam explosion, steam refining, or a combination thereof. As previous studies have shown, the effectiveness of adhesive removal and the impact adhesive removal through pretreatment has on MDF properties depend on various factors including the wood species, the recycled/virgin fiber ratio, and the recycling method used (see Table 1). Savov et al. [60] found the optimal hydrothermal hydrolysis conditions to recover MDF waste are 121 °C for 30 min, which made it possible to reduce the formaldehyde emissions of MDF panels produced entirely from recycled fibers by 41% but significantly affected the panel’s bending strength, IB strength, and thickness swelling. Gürsoy and Ayrilmis [61] showed that adding 7.5% kraft lignin can improve the properties and reduce the formaldehyde emissions of MDF panels made from 10% recycled fibers that have undergone no pretreatment to remove cured UF resin. One promising approach for MDF fiber recovery is a multi-process method that involves using hydrolysis and steam explosion to optimize resin removal, defibration, and water consumption. This method has successfully removed over 80% of the nitrogen while yielding 67% recycled fibers with similar morphological features as virgin fibers [52]. Hagel and Saake [62] proposed to use fractionation by steam refining and acid hydrolysis to recycle MDF waste. This method removed around 80% of the nitrogen and produced more than 70% fibers that are reusable in packaging paper or MDF, plus an extract containing up to 30% carbohydrates. Another innovative process involves incorporating recycled fibers from surface-laminated MDF waste in the core layer of three-layer MDF and combining hydrolysis and extrusion. This process recovered surface-laminated MDF waste to produce new panels with a minimum of 20% recycled fibers and properties comparable to or better than those of virgin fiber panels [63]. On an industrial scale, the European company Unilin Panels has pioneered a method of recovering MDF waste to produce new MDF panels. It involves using steam and high-pressure heat treatment to release the resinated fibers for reuse. The technology was initially tested on panel waste generated within the plant, will soon be tested on post-consumer MDF waste, and could potentially make it possible to replace up to 25% of virgin fibers with recycled fibers [64].

**Table 1:** Summary of recent studies on using post-consumer wood in WBCP manufacturing

New Panel Type	Recycled Material	Recycling Technology	Purpose of the Study	Main Findings | Highlights	Reference
PB	Plywood	Mechanical recycling	To test using different proportions of recycled particles from residual logs, veneers, and plywood waste to manufacture PB	The mechanical properties and thickness swelling of the panels made from residual logs and veneer waste were comparable to those of panels made from virgin particles. The panels made from plywood waste had unsatisfactory mechanical properties due to the presence of residual phenol–formaldehyde (PF) resin	[55]
	PB	Mechanical recycling in a cascade approach	To explore the effect re-milling PB waste (subjecting it to two recycling rounds) on particle characteristics and panel properties	PB produced from the second round of recycling had considerably worse mechanical properties and higher formaldehyde and VOC emissions than PB produced from the first round of recycling. In a cascade recycling approach, the proportion of PB that can be recycled in the next cycle is considerably reduced	[56]
	Surface-laminated PB	Hot water soaking and thermal hydrolysis	To investigate the effect thermal hydrolysis at 100–140 °C for 20, 60, or 100 min has on PBs' physical and mechanical properties, and nitrogen content. Hot water soaking was used for laminate removal	Recycled particles treated at 140 °C for 20 min had the least nitrogen content and about 65% of UF resin removed. Under these conditions, PB made from 100% recycled particles showed good physical and mechanical properties	[59]
MDF	MDF	Hydrothermal hydrolysis	To assess the effect hydrolysis at 121 °C and 134 °C for 30, 45, or 60 min has on the physical and mechanical properties of MDF bonded with MUF resin	On average, formaldehyde emissions decreased by 35% for all treatments. Hydrolysis reduced the MOR and IB strength by 34% and 31%, respectively. Thickness swelling decreased by 18% under less severe conditions (121 °C, 30 min) due to irregularly shaped structures and a low fiber slenderness ratio. The panels did not meet European standards	[60]
	MDF	Acid hydrolysis, thermal hydrolysis, and medium wave frequency	To explore recycling methods for fiberboard made from 100% beech or a 70:30 blend of beech and pine fibers: acid hydrolysis (phosphoric, formic acid), thermal hydrolysis, and medium wave frequency using a microwave device	All methods negatively impacted the bending strength, IB strength, and water absorption of MDF panels bonded with UF resin. The impact on panel properties varied depending on the wood species and pretreatment method used. The panels made with beech and pine fibers that underwent acid hydrolysis or thermal hydrolysis had lower bending and IB strength values than those made with 100% beech fibers	[65]
	MDF	Shredding and steaming without pretreatment to remove UF resin	To determine the effect adding 2.5 to 7.5% kraft lignin has on physical and mechanical properties and formaldehyde emissions of MDF made from recycled and virgin fiber blends (10–30% recycled fiber). Panels were bonded with UF resin	Untreated recycled fibers reduced panel strength, thickness swelling, and water absorption, while recycled fiber content increased formaldehyde emissions. However, adding kraft lignin improved these properties and reducedformaldehyde emissions. The panels containing 7.5% lignin and 10% recycled fiber performed better than the panels made entirely from virgin fibers	[61]
	MDF	Hydrothermal hydrolysis → steam explosion	To evaluate fiber yield, fiber morphology, and resin removal after hydrolysis (180 °C, 15 min) and steam explosion at 180–210 °C and 1.6–2.8 MPa of pressure for 5–20 min with and without additional water	Over 80% the nitrogen was removed from the UF resin, and 67% of the recycled fibers were morphologically similar to virgin fibers. The optimal steam explosion conditions were 180 °C and 1.6 MPa pressure for 20 min, without additional water	[52]
	MDF	Steam refining → acid hydrolysis	To assess MDF waste fractionation by steam refining and acid hydrolysis. Steam treatments varied from 150 to 190 °C for 10 and 20 min	Fiber yield decreased as treatment severity increased. About 20% of the nitrogen remained on fibers regardless of the treatment severity, while 70–80% of it was removed depending on the severity. The nitrogen content in the extract decreased with treatment severity as it degraded into volatile products and passed from the extract to the gas phase	[62]
	Surface-laminated MDF	Shredding and a patent-pending fiber recovery system (hydrolysis → extrusion)	To explore recycling fibers from three types of surface-laminated MDF waste: low-pressure laminate (LPL), polyethylene terephthalate, and polyester coating – and using the recycled fibers in the core layer of three-layered MDF panels in proportions of 10%, 20%, or 30%	The recycled fibers were longer but had more fines than virgin fibers. The physical and mechanical properties of MDF made from LPL-MDF waste were similar to or better than those of MDF made from virgin fibers. Formaldehyde emissions decreased as the recycled fiber content increased, likely due to melamine being released during hot pressing, which can scavenge formaldehyde. At least 20% recycled fiber from LPL-MDF waste can be used in MDF panels	[63]
OSB	Unsorted wood	Mechanical recycling	To test replacing the core layer strands of randomly selected OSB with contaminated wood waste particles. Four levels of recycled particles were used: 0, 25, 50, and 100%. PF resin was used	There was considerable density variation within panels and between core layer compositions. The MOE and MOR decreased as the proportion of recycled particles increased. The mechanical properties other than thickness swelling and water absorption met the requirements set out in CSA standard O437	[66]

When it comes to OSB, there are few studies on recycling OSB waste or using recycled wood to produce new panels, since OSB is made from round wood and has a longer lifecycle than PB or MDF and therefore generates less waste. Schild et al. [66] focused on manufacturing OSB panels with a core layer made of contaminated wood waste particles. They demonstrated that it is feasible to produce OSB with a core layer composed of fully recycled particles that meets the requirements set out in CSA standard O437-93. However, there was considerable density variation within and between panels due to a lack of wood waste sorting.

Finally, wood waste treated with hazardous chemicals such as halogens and heavy metals must be appropriately decontaminated before it can be disposed of or reused (see Fig. 2). Bioremediation offers a sustainable and environmentally friendly alternative to conventional chemical treatments without generating secondary contaminants. Decontaminated wood can be reused for various applications, including manufacturing WBCPs, wood-plastic composites, or wood-cement composites, or even being converted into biocrude and biochar via pyrolysis [53].

## New Product Advances: Key Materials and Properties

### Lightweight Panels

The term”lightweight panels” refers to a variety of material combinations designated to create products lighter than conventional WBCPs while maintaining their functionality and practicality. They are versatile and have many applications in construction, furniture, interior design, and packaging. Their main advantage is their high strength-to-weight ratio, which benefits manufacturers and consumers by enabling better resource utilization, reducing the amount of raw material needed and transportation costs, and meeting consumer demand for lightweight, easy-to-handle, and flat-packed products [67]. Lightweight panels have been studied for years and are not a recent innovation. Nevertheless, we present an overview of the latest developments in lightweight panel manufacturing and outline the key materials used, the challenges encountered, and the main findings reported (see Table 2). Lightweight panels can have various compositions, such as sandwich, single-layer, and multi-layer panels. Sandwich panels comprise thin, rigid surface layers (skins) and a thicker core layer made of a lightweight element such as cellulose paper or cardboard honeycomb, or rigid foam [68–70]. Several commercial versions of these products are available on the market, such as PANOLITE™, a sandwich panel made of a paper honeycomb core (or recycled wood panel core) that can be combined with different skins such as PB or MDF [71]. Sandwich panels are commonly made by assembling layers using discontinuous systems. However, recent research has focused on one-step processes, which enable hybrid panels to be produced using the hot presses that are already in use in the WBCP industry (see [69, 70]). In these processes, the mat is composed of surface layers of wood particles and an expandable core layer that is triggered by temperature during hot pressing. In single-layer and multi-layer panels, foaming materials are incorporated into the adhesive or mixed with wood particles during mat formation [72, 73]. A commercial example is BASF's Kaurit® Light three-layer PB that is produced in one-step for furniture purposes. This PB features a core layer of expanded polystyrene mixed with wood particles and is 30% lighter than conventional PB [74].

**Table 2:** A non-exhaustive list of recent studies on lightweight panels manufactured using different materials and designs

Panel Type	Key Material	Purpose of the Study	Main Findings | Highlights	Reference
PB	Sandwich panels with a rigid foam core	To assess the impact blowing agent (hydrofluorocarbon) concentration has on foam features and the performance of sandwich panels with PB skins and a rigid polyurethane foam core	Foam cell density increased considerably with a blowing agent concentration of 4.5% or higher. Increasing the blowing agent concentration improved bending properties, IB strength, thickness swelling, and water absorption but decreased the edge screw withdrawal resistance. Foam core sandwich panels with desirable properties can be produced by adjusting the blowing agent concentration	[70]
	Foamable sour cassava starch adhesive	To evaluate one-layer lightweight PB manufactured with foamable sour cassava starch adhesive under various pressing conditions	The platen temperature affected starch gelatinization and the foam structure. The foam structure dissipated at platen temperature < 150 °C, resulting in denser panels. Starch gelatinization at 190 °C produced less-dense PBs. Longer pressing times improve foam expansion due to better heat transfer but can lead to a blowout by increasing the internal vapor pressure. The best performance was achieved by pressing at 190 °C for 60 s with a hold time of 540 s. IB strength met the CEN/TS 16368 specifications for lightweight PB (type LP2)	[72]
	Flax shives	To evaluate the physical, mechanical, thermal, and flame-resistance properties of lightweight flax PBs bonded with lignosulfonate or semi-bio-based epoxy	The lightweight panels bonded with lignosulfonate had better bending properties and fire resistance than those bonded with epoxy resin. However, the epoxy-based panels had better dimensional stability and good insulating properties. The flax boards met the minimum requirements set out in standard NF EN 15197 for non-load-bearing applications in dry conditions (type FB2)	[75]
	Hemp shives	To assess the physical and mechanical properties of lightweight PB made from hemp and bonded with UF resin	Three-layer PBs were made with 10% or 25% hemp shives in the surface, the core, or both layers. PBs with 25% hemp shives in the core or the surface and core had similar mechanical properties as conventional PBs and exhibited less thickness swelling	[76]
	Hemp hurd (inner core)	To assess the effect hemp hurd particle size and resin type have on the IB strength of one-layer ultra-low-density PB	Panels were produced with various particle sizes (coarse, medium, fine) and resin types (methylene diphenyl diisocyanate (MDI), phenol-resorcinol–formaldehyde, or bisphenol A-free bio-epoxy). The panels bonded with MDI resin exhibited greater IB strength than those bonded with the other resins. Several MDI-based panel variants met the minimum IB strength requirement of 0.30 MPa set out in the applicable Australian standard. The coarse-particle panels had the highest IB strength, which decreased with the addition of fines	[77]
MDF	Sandwich panels with a paper honeycomb core	To optimize the structure of MDF skins and the paper honeycomb core in sandwich panels for maximum load capacity in three-point bending tests	Sandwich panels with different skin and core thicknesses, honeycomb directions, and span distances were evaluated for their failure load. The skins and honeycomb core were joined with a polyvinyl acetate adhesive. The sandwich panels with a perpendicular honeycomb had higher failure load values than those with a parallel honeycomb, regardless of skin or core thickness. The failure load decreased as the span distance increased. Reducing the skin thickness (strength) or reinforcing the core can optimize sandwich panel performance. Further studies are required to adjust skin and core thickness for optimal results	[68]
	Sandwich panels with a foam core	To develop foam-core fiberboard in a one-step process and assess the effect expandable polystyrene bead diameter and surface layer thickness have on panel properties	Increasing the polystyrene bead diameter adversely affected bending properties and edge screw withdrawal resistance but improved thickness swelling and water absorption. Increasing the surface-layer thickness improved the MOR, MOE, and face screw withdrawal resistance but worsened thickness swelling, water absorption, and edge screw withdrawal resistance. IB strength remained unchanged for all treatments. Sandwich panels with 3 mm skins and 0.5 mm polystyrene beads in the core met the specifications set out in European standard EN 622–5 and were almost 55% less dense than conventional MDF	[69]
	Kenaf core fiber	To produce lightweight fiberboard from co-refined 50:50 rubberwood-kenaf fiber and evaluate the effect panel density and UF resin content have on panel properties. The fiberboard was compared with commercial MDF	Panel density had a greater impact on lightweight panel performance than UF resin content. The lightweight 550 kg/m^3^ panels had superior bending properties than the 720 kg/m^3^ commercial MDF. The IB results were inconsistent. The thickness swelling of the lightweight panels ranged from 12 to 18% depending on panel density and resin content, which is higher than that of commercial MDF but still meets the specifications set out in standard EN 622–5 for ultra-light MDF in dry conditions (UL1-MDF)	[78]
OSB	Low-density kiri wood	To evaluate the effect wood density has on the physical and mechanical properties of lightweight (non-oriented) strand board	Lightweight panels made from low-, medium-, and high-density woods (kiri, pine, and beech, respectively) were compared. The kiri-based panels had a higher compaction ratio, a more optimal density profile, and superior bending and IB properties than the pine and beech panels but exhibited greater thickness swelling	[79]
	CNF	To explore reducing the density of OSB by partially substituting pMDI with unbleached CNF	Replacing pMDI with 6% unbleached CNF allowed the reduction of the OSB density by 0.09 and 0.08 g/cm^3^ for equivalent MOR and MOE performance. All other properties except IB strength remained unchanged. Spraying CNF led to clogging issues. Additional research is required to improve the spraying procedure consistency	[80]

The conventional foam materials used for lightweight panels are polystyrene, polyurethane, phenolic, and polyolefin, which are petroleum-derived, combustible, and non-biodegradable [69, 70, 73, 81]. Bio-foams that are based on proteins, lignin, starch, and tannin have been studied to replace them, although only a few have been tested in PB and MDF manufacturing [72, 82–84]. For example, Zhou et al. [85] showed that lightweight sandwich panels made with a rigid skin (wood, plywood, MDF, or fiber composite) and a tannin-furanic rigid foam core are a feasible and more eco-friendly option than petroleum-based foams. However, using tannin-based foams comes with some challenges, such as impurities resulting from the extraction process and the heterogeneous characteristics of tannins [84]. Monteiro et al. [72] investigated the impact that hot-pressing parameters have on foam formation and the performance of PB made with foamable sour cassava starch. Their main findings are summarized in Table 2. Foaming materials used for the core layer of panels must have the following key features: be expandable, be able to expand at a specific activation temperature (~ 100 °C), be able to maintain a solid shape for mat formation prior to expansion, be non-sticky for uniform felting or scattering, and be able to resist pressure while in an unexpanded state [70]. These features complicate bio-foam selection and compatibility with the continuous systems used in WBCP manufacturing, particularly in the mat formation and hot-pressing steps.

Bio-fibers sourced from non-wood lignocellulosic material, such as kenaf, flax, and hemp, have also been explored as more sustainable alternatives to non-renewable lightweight materials in lightweight panels (see Table 2) [75–78]. These fibers are longer and have a favorable chemical composition, which means they are able to give panels exceptional strength while reducing their weight. Long fibers offer a higher aspect (length-to-diameter) ratio, which is particularly advantageous for fiberboard, as it reduces the amount of adhesive required and increases uniform adhesive distribution. Also, long fibers tend to lie horizontally during mat formation, which improve bending properties. Compared to wood, these fibers contain more cellulose. For example, flax contains 65–85% cellulose, and hemp 60–68%, while hardwoods contain 42–49%, and softwoods, 42–51% [86]. Since cellulose provides strength and dimensional stability, flax and hemp fibers are expected to produce lighter PB with superior bending strength, thickness swelling, and water absorption properties [87]. The main challenges when developing lightweight panels are IB strength and ensuring connectors such as screws and bolts are properly anchored in the core layer [87, 88].

When it comes to OSB, which is intended for structural applications, the options for producing lighter panels without compromising safety are limited. Therefore, improving the manufacturing process has been the strategy used to enhance the strength-to-density ratio of OSB. This can be done by optimizing strand geometry and alignment, using low-density wood species to improve the compaction ratio, or improving horizontal density uniformity to minimize weak points associated with failure during concentrated static load testing [79, 89, 90]. Also, a recent study showed that partially replacing polymeric 4,4-diphenylmethane diisocyanate (pMDI) with cellulose nanofiber (CNF) reinforced OSB and gave it bending properties equivalent to those of conventional panels while reducing its overall density and weight (see Table 2) [80].

### Fire-Resistant Panels

WBCPs used for certain building applications must be highly fire-resistant to ensure the safety of occupants and meet building code requirements. However, wood is inherently combustible and undergoes a complex physical and chemical combustion process depending on its chemical components, moisture content, density, and surface treatment [91]. For WBCP in particular, other factors, such as a panel’s structure, moisture content, density, and thickness, the adhesive used, and the presence of additives affect their combustibility. Material flammability is commonly associated with ignitability, flame-spread rate, and heat release and is usually measured by the limiting oxygen index, flame spread index, or cone calorimetry [92].

FRs are used in surface coatings and adhesives and for wood impregnation and encapsulation [91]. They are classified as either halogenated or non-halogenated. Halogenated FRs are low-cost, effective, and widely available in the market, but they are harmful to the environment and human health due to their persistent, bioaccumulative, and mobile nature [93, 94]. Moreover, panels containing halogens are challenging to recycle, as previously discussed. For these reasons, several halogenated-brominated FRs have been banned in Europe under the REACH (Registration, Evaluation, Authorization, and Restriction of Chemicals) Regulation or the Stockholm Convention on Persistent Organic Pollutants (POPs). Countries such as China, Japan, India, and the US are also taking measures to regulate their use [94]. Halogen-free FRs encompass a wide range of products, such as inorganic systems (phosphorus, boron, nitrogen, and metal hydroxide), organic systems (organophosphorus-like ammonium polyphosphate), nanofillers, nanomaterials, and intumescents, the latter of which form a protective char layer on the surface of a material when exposed to heat [95, 96]. Boron and phosphorus compounds, and combinations thereof, are commonly used for fire protection of WBCPs [97–99]. Boric acid is on the Candidate List of substances subject to the REACH Regulation due to its reproductive toxicity [92]. Phosphorus compounds, despite being considered eco-friendly due to their low toxicity, halogen-free, and non-corrosive nature, generate smoke and CO emissions during combustion [81, 100]. Also, some inorganic FRs are water-soluble and easily leached from substrates upon exposure to moisture, in addition to adversely affecting panel properties [101]. Nanomaterials such as nanoclays (nano-montmorillonite, nano-wollastonite), nano-oxides (nano-SiO_2_, nano-TiO_2_, nano-ZnO), and carbon-based nanoparticles (carbon nanotubes, graphite, expandable graphite, graphene) have been investigated as alternative FRs for WBCPs. These materials can offer oxygen and heat barrier properties, improve char formation, and reduce CO and CO_2_ production during combustion. However, their toxicity levels for human health and the environment are unclear and depend on the nature and properties of the nanoparticle [96]. Nanoclays are reported to be non-toxic and harmless [102], while metal-based nanoparticles are considered potentially toxic elements depending on their physical and chemical properties (concentration, size, surface properties, surface area, porosity, charge, and agglomeration) [103]. Other FR systems include innovative materials like metal–organic frameworks (MOFs), molecular sieves, and polyelectrolyte complexes (PECs). MOF-based systems have been shown to enhance the effectiveness of organic and inorganic FRs, and to suppress the smoke produced due to their high specific surface area and ability to absorb pyrolysis gases [104]. Recent studies have also shown that zeolite-based molecular sieves can absorb VOCs and form a stable char layer that reduces combustion and gas release. According to Li et al. [105], MDF made with zeolite-based FR can effectively separate pyrolysis gas and air, to avoid further combustion and thus help to reduce heat release, smoke production, and CO emissions. PECs have recently been studied to serve as eco-friendly, highly flame-retardant coatings for wood products. PEC-treated OSB exhibited less smoke and heat release, a longer ignition time, and more char residue. However, the authors found that PEC-treated OSB undergoes leaching, leading to a 3% total weight loss after a 24-h soak, reducing flame retardancy. They propose conducting further research on the cross-linking of wood cellulose and PEC components to enhance the PEC treatment’s durability [106].

Various natural substances such as saccharides (cellulose, hemicelluloses, starch, and chitosan), lignin, DNA, proteins (e.g., casein, which is a co-product of skim milk production), phytic acid (sourced from oilseeds, nuts, and cereal), and vegetable oils have been explored as promising FR alternatives. These substances have been found to exhibit noteworthy charring capacity for intumescent FRs [107]. For example, Lin et al. [100] showed that a combination of phytic acid and sodium silicate can effectively enhance the flame retardancy of PBs without compromising their physical and mechanical properties. They showed that adding 12% phytic acid improved the fire retardance of PBs by reducing the amount of heat and smoke generated and increasing the flashover time (therefore reducing the risk of fire propagation). Cho et al. [108] demonstrated that a phosphorylated chitosan-based coating reduces the total and peak heat release rates by over 96% and increases the amount of char residue. Table 3 summarizes some emerging materials that are used for fire protection of WBCPs and are considered non-toxic to human health and the environment.

**Table 3:** A non-exhaustive list of emerging materials used for fire protection of WBCPs

Panel Type	Fire Retardant System	Purpose of the Study	Main Findings | Highlights	Reference
PB	Phosphorylated cellulose microfiber (P-CMF)	To improve the fire resistance of PB using P-CMF from giant reed (GR) plants	Raw GR fiber, pure CMF, and P-CMF were mixed with UF resin at loads of 5%, 7.5%, and 10% (based on the UF solid weight). Cellulose fibers improved panel mechanical properties and reduced formaldehyde emissions. P-CMF at 7.5% improved panel fire resistance by, reducing the flame spread speed by 65% and the after-flame time by 40%	[109]
	Phytic acid–silica-based	To improve the fire resistance of PB by treating wood particles with an aqueous phytic acid and sodium silicate solution	Increasing the phytic acid content increased the limiting oxygen index value (for better fire resistance) and char formation, and reduced heat release, smoke production, and mass loss. The phosphate group of phytic acid reduces heat release and acts as a scavenger, which favors char residue and the formation of a protective insulating layer	[100]
	Lignin-based	To determine the effect kraft lignin and lignosulfonates have on the flammability and physical and mechanical properties of PB made from areca agricultural waste and bonded with UF resin	Flammability was tested in accordance with the standard UL-94. Panels made with lignosulfonate that underwent five washing treatments exhibited the least weight loss after the burning test. Both kraft lignin and lignosulfonate improved the MOR	[110]
PB | Wet-process fiberboard	Lignocellulosic nanofiber (LCNF) from date palm waste	To test using LCNF made from date palm waste as a binder to improve the properties and fire retardance of panels made from rapeseed stalk waste	Fiberboard made with 2% LCNF and PB made with 5% LCNF met the flammability requirements for commercial buildings. Increasing the LCNF content increased the flame propagation time. Fiberboard made with 15% LCNF had higher specific MOE, MOR, and IB values than the commercial panel reference. LCNF improved the thickness swelling and water absorption of the fiberboards, which exhibited better performance than the commercial reference	[111]
MDF	Nano-wollastonite	To find the optimal nano-wollastonite level	Nano-wollastonite improved the thermal conductivity and fire resistance of MDF panels. Increasing the nano-wollastonite content from 2 to 8% significantly increased the ignition time and glow time and decreased the burn duration and burnt area	[102]
	Casein-based	To develop fire-resistant MDF panels by adding casein using various loading and application methods	Casein slightly reduced the heat release rate of MDF panels and formed a more compact char layer. It also improved the bending strength of MDF by 48%	[112]
	Zeolite-based molecular sieves	To improve the flame resistance of MDF by treating wood fibers with a zeolite composed of water glass (30% Na_2_SiO_3_) and aluminum sulfate salt (Al_2_(SO_4_)_3_) as a precursor	The zeolite-based FR produced a stable char layer that prevented smoke production and reduced combustion and CO and CO_2_ release. The treatment decreased the panels’ formaldehyde emissions and slightly impacted their physical and mechanical properties, but their properties still met all the requirements set out in the applicable standards	[105]
OSB	MOFs	To develop an intumescent FR system (APz@ZIF67) by combining Co-MOF (ZIF67) and piperazine-modified ammonium polyphosphate (APz)	APz@ZIF67 increased the mechanical properties and flame retardance of panels. The limiting oxygen index increased by 71.37%, while the heat-release and total smoke rates decreased by 48.61% and 43.78%, respectively. However, APz@ZIF67 also decreased IB and increased thickness swelling	[104]
	Boron-based PEC coating	To assess a PEC coating composed of sodium polyborate and polyethylenimine for fire protection of OSB	The total smoke release, peak heat release, and total heat release decreased by 79%, 18%, and 21%, respectively. PEC treatment also increased the ignition time by 18% and char residue by 35%	[106]

### Electrically Conductive and Electromagnetic Shielding Panels

Adding conductive polymers or metals to wood particles, fibers, adhesives, or coatings can give WBCPs electrical properties that can enable innovative applications such as cable-free electrical connections, electromagnetic shielding, and intelligent fire, occupancy, or flooding detection using temperature, pressure, or liquid sensors [113–116].

The growing trend of integrating electronics into furniture and advancements in cable-free connection technologies have led to the development of electrically conductive MDF using carbon-based fiber (CF) [113]. Tschannen et al. [113] explored the effect of CF content, blending process (wet or dry), and resin type (UF or pMDI) on the electrical, physical, and mechanical properties of MDF panels. The blending process improved electrical conductivity up to 230 S/m but reduced mechanical properties. UF-bonded panels had superior electrical properties. As a proof of concept, a cube-shaped furniture prototype was also prepared using five-layer MDF that was bonded with UF resin and contained 5% CF. Lighting inside the prototype was successfully tested using LED pins.

Another innovative approach is developing MDF with electromagnetic shielding properties [114, 115]. Electromagnetic shielding materials can help reduce the harmful effects that electromagnetic emissions from electronic devices have on the environment and human health or protect electronic devices from electromagnetic interference in specific applications [115, 117]. Pourjafar et al. [114] studied the effect varying the proportion of CF has on the electromagnetic shielding effectiveness of MDF bonded with UF-isocyanate resin. Their results indicated that at high proportions, CF reduces the physical and mechanical properties of panels. However, at low CF proportions, panel properties met all the requirements set out in applicable standards while exhibiting high electromagnetic shielding effectiveness. These studies show that manufacturing CF-containing panels can be challenging due to issues related to uniform distribution, blending methods, panel properties, and the cost of CF. CF from renewable sources could also improve sustainability.

### Antimicrobial Laminate Surfaces

PB and MDF are generally covered with decorative paper impregnated with thermosetting resin, usually melamine–formaldehyde resin. Such laminates are decorative and functional – they act as a moisture barrier and reduce formaldehyde and other VOC emissions from the panels. Although laminate surfaces can offer some level of resistance against microorganisms, they have no biocidal activity. To this end, organic or inorganic additives can be combined with the thermosetting resin to create antimicrobial laminates that are capable of inhibiting microorganism growth (biostatic) or killing microorganisms (biocidal) [118]. Antimicrobial additives must be chemically and physically inert to the resin and have no impact on the properties of the laminate [14]. In North America, antimicrobial technologies for hard surfaces must meet health and safety requirements set out by the US Environmental Protection Agency (EPA) or Health Canada. Laminates’ antibacterial ability is measured in accordance with ISO standard 22,196:2011 against two pathogenic bacteria strains: gram-positive *Staphylococcus aureus* and gram-negative *Escherichia coli* [119]. Other bacteria strains can be tested using the same procedure.

Inorganic nanoparticles are frequently utilized to produce laminates with antibacterial and antimold properties for PB and MDF. Adding silver nanoparticles (Ag-NPs) to the melamine-laminated surfaces of PB was found to reduce the growth of *E. coli* by 100%, *S. aureus* by 53.7%, and *Penicillium brevicompactum* mold by 62.5%. The addition of Ag-NPs did not affect the surfaces' resistance to harsh chemicals or dry heat but did result in a 12% reduction in their abrasion resistance [14]. Yontar et al. [15] studied the green synthesis of Ag-NPs using *Cannabis sativa* seed extract for melamine-laminated MDF surfaces. Green synthesis of Ag-NPs involves using plant extracts, offering cost and environmental benefits by minimizing the use of toxic chemicals (see [120]).The authors reported that *Cannabis sativa* contains high levels of secondary metabolites that reduce the amount of Ag^+^ ions in aqueous solutions, which enables Ag-NP synthesis and prevents nanoparticle agglomeration. Their results showed that their green-synthesized Ag-NPs were 1.5 times more effective against gram-negative *E. coli* than gram-positive *S. aureus* due to stronger electrostatic interaction with the positively charged Ag-NPs. The laminate containing 1% Ag-NPs was almost twice as abrasion and scratch resistant as the standard value of 150 cycles and 1.75 N, respectively, while remaining stain-resistant [15]. He et al. [121] studied the green synthesis of Ag-MOFs for developing antibacterial melamine-impregnated paper. Ag-MOFs act as reservoirs, release Ag^+^ ions over time, and are highly effective against *E. coli* and *S. aureus*. Furthermore, Ag-MOFs are compatible with MF resin, exhibit good thermal stability, and meet the requirements for hot-pressing melamine-impregnated paper. Silver-based antimicrobial solutions are commercially available for high-pressure laminate impregnated with phenolic or melamine resin. One such example is the silver ion technology from Microban^©^, called SilverShield®, which is approved for food-contact use and has been proven to be non-leaching and effective against a wide range of harmful bacteria such as *Salmonella enterica*, *E. coli*, methicillin-resistant *Staphylococcus aureus* (MRSA) and vancomycin-resistant enterococci (VRE) [122]. Although these antimicrobial laminates have advantages, their potential toxicity, hazardous effects on the environment (see [99]), and recyclability must be assessed. Using natural compounds that have antimicrobial properties, such as chitosan, may be considered a more sustainable approach [123].

Other advanced surface technologies involve incorporating nanomaterials into melamine–formaldehyde resin to improve the mechanical properties, thermal stability, and fire retardancy of melamine-impregnated papers, and to reduce the formaldehyde and VOC emissions of coated panels [124]. For instance, coating PB and MDF panels with melamine-impregnated paper containing TiO_2_ or montmorillonite nanoparticles reduced the panels’ formaldehyde emissions by 22–36.6% and total VOC emissions by 22.6–25.6% [125]. Another approach involves plasma-pretreating panel surfaces, which can enhance coated panels' abrasion and scratch resistance [126, 127]. Air plasma-pretreating PB using a dielectric barrier discharge (DBD) device at atmospheric pressure has been shown to have a positive impact on the abrasion resistance of waterborne acrylic coatings [126]. Also, DBD plasma treatment modified the wettability and surface roughness of the PB and MDF panels, and improved the epoxy coating’s performance (adhesion strength and scratch resistance), especially for MDF [127]. More research is needed to assess how plasma treatments impact the performance of melamine-impregnated paper.

## Conclusions and Perspectives

Climate change and competition from the energy sector threaten current sources of fiber supply for panel manufacturing in some locations. Logging residues are available in large quantities and underutilized in North America. From a technological perspective, using logging residues in the panel industry requires that residues be properly classified, suitable fragmentation technologies be developed, and panel fabrication parameters be adjusted. Fire-damaged wood may also be worth exploring for panel manufacturing, as wildfires are expected to become more frequent and intense due to climate change. Given the scarcity of existing research, further studies are needed to better understand how using fire-damaged wood affects overall panel properties. Although there is growing interest in using recycled wood in panel manufacturing, the industry still faces challenges when it comes to effectively recycling panels, particularly MDF. Overall, the hazardous chemicals in adhesives, coatings, and FRs pose a challenge for recycling panels and creating a more circular economy within the WBCP industry. The volume of panel waste will increase significantly in the coming years as global WBCP production continues to grow. It is therefore of interest to consider the use of new materials to address the issue of recycling panels. Life cycle assessments must be completed for raw material sources to determine the best use paths from an environmental perspective.

The latest breakthroughs in lightweight panels show that developing bio-foams that have all the attributes necessary for use in panel manufacturing processes, and provide the same panel properties as conventional non-renewable foams, is a notable challenge. Natural fibers derived from agricultural residues, like flax and hemp, are widely used as reinforcement for lightweight panels. Still, fiber size, fiber proportion, IB strength, and edge screw withdrawal resistance improvements are needed to ensure all requirements are met.

FRs raise environmental issues due to halogens, which make panel recycling difficult and have long-term environmental impacts. Natural substances like cellulose, nanocellulose, chitosan, lignin, protein, and phytic acid have been shown to exhibit notable intumescent charring capacity when combined with phosphorous FRs, and are promising and more sustainable alternatives to halogenated FRs.

New advances in conductive panels focus on the latest trends in integrating cable-free electronics into furniture, and shielding panels designated to protect the environment or human health from hazardous electromagnetic emissions.

Recent research has focused on antimicrobial laminates containing silver. These laminates have been proven to effectively combat bacteria, mainly gram-negative bacteria, which makes them an ideal option for ensuring high hygiene levels on frequently touched surfaces in hospitals and workplaces. However, it is essential to consider the future recycling potential of laminated panels.

In summary, the following steps are recommended to overcome the challenges and take advantage of the opportunities in WBCP manufacturing:

1. Assess the impact of climate change, wildfires, and competition with other sectors on the fiber supply for panel manufacturing, considering the socio-economic and environmental issues linked to the local or regional context.
2. Explore the economic, environmental, and technological aspects of using logging residues and fire-damaged wood for panel manufacturing with a view to strategic planning.
3. Investigate recycling opportunities, adopt environmentally friendly materials that can facilitate recycling in the future, and carry out life-cycle assessments of raw material sources, processes, and products for eco-responsible decision-making.

## Data Availability

No datasets were generated or analysed during the current study.
